# Epidemiological and clinical characteristics of scrub typhus in northern Fujian, China, from 2015 to 2019

**DOI:** 10.1186/s12879-023-08451-1

**Published:** 2023-07-18

**Authors:** Jin Huang, Kaixiang Deng, Jiawei Chen, Meiquan Zhang

**Affiliations:** 1 Department of Infectious Diseases, The Second Affiliated Hospital of Fujian University of Traditional Chinese Medicine, Wusi Road, Fuzhou, China; 2 Department of Traditional Chinese Medicine, First Hospital of Nanping City, Nanping, China; 3 Department of Pulmonary and Critical Care Medicine, Fujian Provincial Geriatric Hospital, Fuzhou, China

**Keywords:** Scrub typhus, Clinical characteristics, Epidemiology, Orientia tsutsugamushi, Public health

## Abstract

**Background:**

This study aimed to analyze the epidemiological and clinical characteristics of scrub typhus in northern Fujian Province on the southeast coast of China.

**Methods:**

A retrospective analysis was performed on 303 patients with scrub typhus admitted to the First Hospital of Nanping City, Fujian Province, from January 2015 to December 2019. The epidemic characteristics were analyzed, such as the annual number of cases, age distribution, sex distribution, and seasonal distribution in each region. The patient's clinical manifestations, signs, complications, auxiliary examinations, and prognosis were analyzed.

**Results:**

From 2015 to 2019, the age distribution of scrub typhus cases was mainly concentrated in 40–49 y (17.16%), 50–59 y (24.09%), and 60–69 y (26.73%). There were no sex differences among the patients. 68.98% of the cases were concentrated in rural areas, with farmers having the highest proportion. However, this study compared prognostic factors in the cured and uncured groups, and found significant differences in non-farmer occupation and diagnosis time ≥ 8 days. Scrub typhus showed two peaks north of Fujian; the prominent peak was from June to July, and the other slight rise was from October to November. The SDE plot showed that the cases were mainly concentrated in Yanping, Shunchang, Zhenghe, and Songxi counties. The number of cases in hilly and mountainous areas was higher than in plain areas. The main diagnostic methods in this area are based on specific eschar and epidemiology, while the positive rate of the Weil-Felix test is low.

**Conclusions:**

The results of this study can guide primary care institutions to improve the level of diagnosis and treatment of scrub typhus and take effective public health intervention measures in endemic areas.

## Background

Scrub typhus is one of the world's most prevalent and clinically significant rickettsial infections. It is a vector-borne zoonotic sickness caused by the bacterium Orientia tsutsugamushi. Orientia tsutsugamushi is an intracellular gram-negative bacterium that requires host cells and is transmitted to humans through the bite of chigger larvae [1]. The clinical manifestation of scrub typhus is characterized by fever, headache, chills, myalgia, and typical eschar or rash at the location of the bite, which can lead to multiple organ failure and even death in some cases [2]. It is estimated that scrub typhus threatens one billion people globally and causes at least one million clinical issues annually in Asia–Pacific [3]. China has a long history of scrub typhus and is a crucial epidemic area. In recent years, the prevalence of scrub typhus has increased significantly in many regions of China, especially in rural areas [4]. Local cases have been reported in 29 provinces except for Ningxia Hui Autonomous Region and Shanghai, increasing annually. According to latitude, scrub typhus can be divided into a southern, transitional, northern, and plateau climate epidemic zone [5, 6]. According to the season of onset, scrub typhus in China can be divided into three types: summer, autumn, and winter. There are differences in the spatial and temporal distribution of epidemics in different provinces [7].

The northern Fujian region of Fujian Province is located on the southeast coast, is the central mountainous area on the southeast coast, and is the main epidemic area for the disease [8]. Rickettsial infection is a multisystem disease that may cause serious complications such as acute hepatic dysfunction, septic shock, acute kidney failure, acute myocarditis, disseminated intravascular coagulation (DIC), respiratory failure, multiple organ failure, or even death. If diagnosis or proper treatment is delayed, mortality rates as high as 35–60% have been reported [9]. The epidemiological characteristics of scrub typhus in different regions of China are still unclear, and the differences in clinical characteristics and temporal and regional distribution require further study. Owing to its variable and nonspecific clinical presentation, weak knowledge, low index of suspicion among doctors, and lack of diagnostic resources, it is typically underdiagnosed in China.

There have been no large-scale clinical reports of scrub typhus in this area. Therefore, a retrospective clinical study of scrub typhus was conducted at the First Hospital of Nanping City, Fujian Province, China. A total of 303 patients with scrub typhus admitted to the First Hospital of Nanping City between January 2015 and December 2019 were enrolled in this study. A detailed understanding of the epidemiological and clinical characteristics of scrub typhus in Fujian Province is needed to develop more effective public health strategies for the occurrence of scrub typhus.

## Methods

### Data collection

This project is jointly completed by the Second People's Hospital of Fujian (Also called "The Second Affiliated Hospital of Fujian University of Traditional Chinese Medicine”) Province and the First Hospital of Nanping City. The First Hospital of Nanping City is the largest tertiary hospital in northern Fujian Province and a designated hospital for treating scrub typhus. This study systematically reviewed scrub typhus patients' epidemiology and clinical characteristics in the Northern Province of Fujian, including sex, age, occupation, clinical symptoms, signs, complications, and prognosis. Clinical data were obtained from medical records of the First Hospital of Nanping City. Data from 303 patients with scrub typhus admitted to the First Hospital of Nanping City, Fujian Province, from January 2015 to December 2019 were included, including 151 males and 152 females, with an age range of (7–85) years. All patients had a history of fieldwork, grass play, or crops. The treatment duration was mainly 1–6 days, with an average of 5–7 days and maximum of 21 days.

### Diagnostic criteria

Scrub typhus cases were classified as probable (clinically diagnosed) or confirmed (laboratory confirmed) according to the Chinese national health authorities' diagnostic criteria and case classification guidelines. Probable cases are diagnosed by experienced local physicians based on epidemiologic exposure (travel to a disease-endemic area and contact with chiggers or rodents < 3 weeks before the onset of illness) and clinical manifestations (such as high fever, lymphadenopathy, skin rash, and eschars or ulcers). Suspected or clinical cases with one of the following laboratory test results were confirmed: a positive outcome in pathogenic isolation, a Weil-Felix OXK agglutination titer ≥ 1:160, a fourfold rise in immunoglobulin G with the indirect immunofluorescent assay, or a positive outcome with nested polymerase chain reaction. All cases of scrub typhus were confirmed according to the diagnostic criteria issued by the Chinese Center for Disease Control and Prevention [10]. This study included 286 patients with clinical diagnoses and 17 patients with laboratory diagnoses.

### Laboratory indicators

In this project, 303 patients with scrub typhus patients were collected for routine blood tests (BRT), biochemical test (Chem-Bio Detection), Weil-Felix test (OXK agglutination titer ≥ 1:160), urine routine, coagulation function, erythrocyte sedimentation rate (ESR), C-reactive protein (CRP), procalcitonin (PCT) and other laboratory indicators.

### Statistical analysis

This study analyzed clinical and laboratory-confirmed scrub typhus in the First Hospital of Nanping from 2015 to 2019. Scrub typhus is a notifiable infectious disease in China. All cases of scrub typhus must be reported to the Chinese Centers for Disease Control and Prevention. We obtained population data from the National Bureau of Statistics of China to calculate incidence rates. According to the sixth national census data, there are 2,645,549 permanent residents in Nanping [11]. GraphPad Prism (version 8.0) was used for statistical analysis and graph generation, and ArcGIS (version 10.8.2) was used for topographic and standard deviation ellipse (SDE) mapping. The SDE was drawn to understand the direction and range of the distribution of cases in northern Fujian. The major and minor half-axes of the ellipse represent the direction and range of data distribution. The directionality of the distribution is more visible the more significant the difference between the major and minor half-axis. The constituent ratio is used to express the proportion and distribution of each component of the disease in the population, generally expressed as a percentage. The chi-square test was used to compare the related factors between the cured and uncured groups. Statistical significant was set at *p* < 0.05.

## Results

### Demographic features

**Fig. 1:**
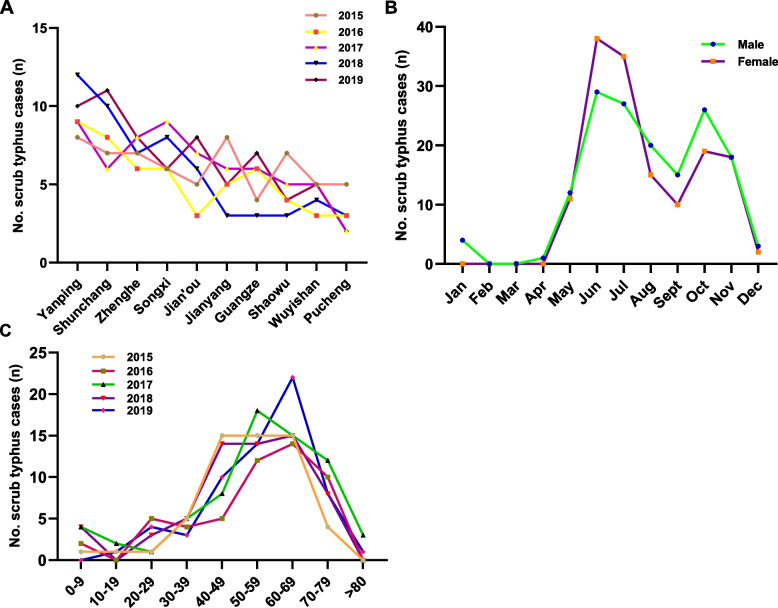
The distribution of scrub typhus patients in different age groups; **A **The number of scrub typhus cases in northern Fujian counties and cities from 2015 to 2019; **B** Sex distribution of the number of scrub typhus cases per month; **C **The number of scrub typhus cases in different age groups from 2015 to 2019

In northern Fujian, the number of scrub typhus patients was higher in Yanping, Shunchang, Zhenghe, and Songxi counties (Fig. 1A). Among 303 patients with scrub typhus, 151 (49.83%) were male, and 152 (50.17%) were female. There was no significant difference in the number of hospitalized patients between males and females (Fig. 1B). Figure 1C shows the distribution of scrub typhus cases in different age groups from 2015 to 2019, with patients mainly concentrated in 40–49 years (17.16%), 50–59 years (24.09%), and 60–69 years (26.73%). Age clustering was related to occupation, and the high-risk population had a more frequent history of field activities, especially farmers (76.57%) who had engaged in agriculture for a long time. Farmers (76.57%) were generally the most affected group, with 68.98% of the cases occurring in rural areas (Table 1).

**Table 1:** Characteristics of reported scrub typhus 303 cases in Nanping, China, 2015–2019

Variable	n	Constituent ratio (%)	Variable	n	Constituent ratio (%)
**Age groups (y)**			**Residence**		
0–9	11	3.63%	Rural	209	68.98%
10–19	4	1.32%	Urban	94	31.02%
20–29	14	4.62%	**Occupation**		
30–39	22	7.26%	Farmer	232	76.57%
40–49	52	17.16%	Worker	21	6.93%
50–59	73	24.09%	Retiree	18	5.94%
60–69	81	26.73%	Student	15	4.95%
70–79	42	13.86%	Anglers	8	2.64%
> 80	4	1.32%	Mountaineer	4	1.32%
**Sex**			Police	2	0.66%
Male	151	49.83%	Teacher	2	0.66%
Female	152	50.17%	Geologist	1	0.33%

### Spatial distribution

**Fig. 2:**
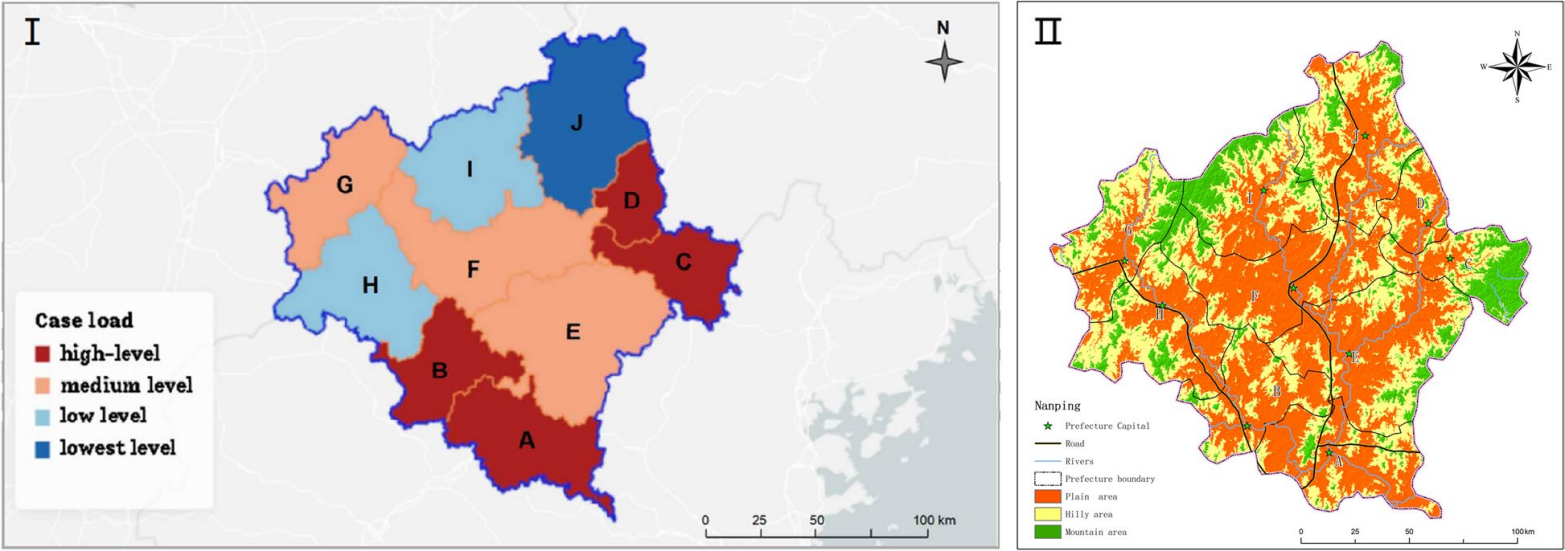
Spatial distribution and geographical morphology of scrub typhus in northern Fujian Province, China, 2015–2019. (A) Yanping District; (B) Shunchang County; (C) Zhenghe County; (D) Songxi County; (E) Jian’ou County-level City; (F) Jianyang District; (G) Guangze County; (H) Shaowu County-level City; (I) Wuyishan County-level City; (J) Pucheng County. (**I**) This project uses Dycharts software (Web version) to draw the administrative region map of northern Fujian Province. The URL is https://dycharts.com/appv2/#/pages/workspace?project=166435759529979334&type=chart&from=old. (**II**) ArcG -IS (version 10.8.2) was used for topographic. The URL is http://t0.tianditu.gov.cn/ter_c/wmts, you need to add the URL to the ArcGIS software to open it

**Fig. 3:**
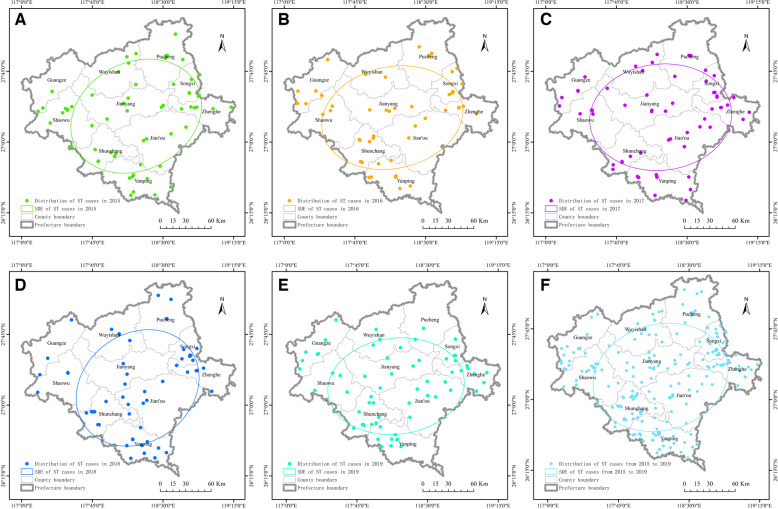
Standard deviation elliptical plot of the spatial distribution of scrub typhus cases from 2015–2019; **A**-**E** The SDE plots of ST cases in each year from 2015 to 2019, respectively; **F** The SDE plot for all cases; (ST) scrub typhus; (SDE) standard deviation ellipse. According to the longitude and latitude of each patient, ArcGIS 10.8.2 software was used to draw the spatial distribution map. The URL is http://t0.tianditu.gov.cn/vec_c/wmts, you need to add the URL to the ArcGIS to open it

The First Hospital of Nanping City is the largest tertiary hospital in northern Fujian and is designated for treating scrub typhus. Three hundred and three scrub typhus patients were from 10 districts (counties/cities) north of Fujian, including Yanping, Jianyang, Shunchang, Zhenghe, Shaowu, Wuyishan, Songxi, Guangze, Pucheng, and Jianou. The extent of the epidemic in each district (county/city) can be indirectly assessed based on the population proportion and number of hospitalized patients. There were 48 Yanping cases, accounting for 15.84% of the total cases. There were 42 cases in Shunchang County, accounting for 13.86% of the total cases. There were 36 cases in Zhenghe County, accounting for 11.88% of the total. There were 35 cases in Songxi County, accounting for 11.55% of the total, and the number of cases in these areas was high. There were 29 cases in Jian'ou City, accounting for 9.57% of the total cases. There were 27 cases in Jianyang District, accounting for 8.91% of the actual cases. There were 26 cases in Guangze County, accounting for 8.58% of the total, and these areas were at a medium level. There were 23 cases in Shaowu City, accounting for 7.59% of the total cases. The number of cases in Wuyishan City was 22, accounting for 7.26% of the total, which is low. There were 15 cases in Pucheng County, accounting for 4.95% of the total, which was the lowest in the region (Fig. 2I). Nanping City is located in the north of Fujian Province, the main terrain is hilly, mountainous, dense forests, shrubs and weeds, mountain streams, rivers all over the upper reaches of the Minjiang River. The external environment is suitable for the reproduction of chigger larval mites. Among the ten counties and cities, Jianyang and Jianou plain areas were relatively broad, and the other counties and cities were mainly hilly and mountain areas (Fig. 2II). The SDE plot results showed that the distribution of scrub typhus in northern Fujian from 2015 to 2019 had a specific direction. The scrub typhus cases in this hospital mainly came from Yanping District, Shunchang County, Zhenghe County, and Songxi County (Fig. 3A-F).

### Temporal and seasonal distribution

**Fig. 4:**
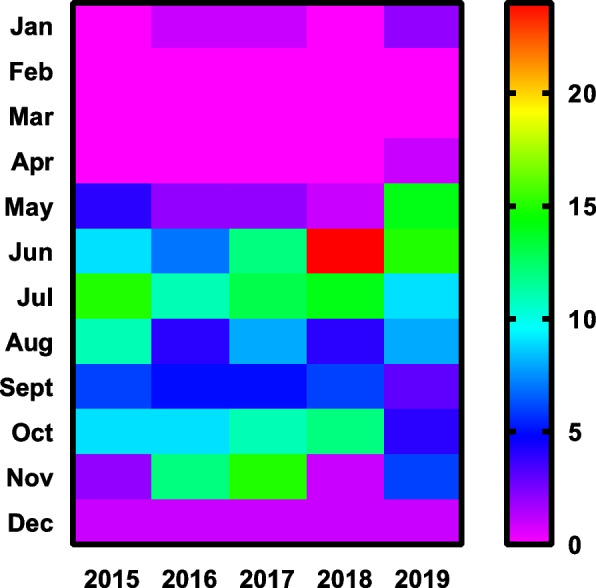
Monthly distribution of scrub typhus patients in northern Fujian Province, China, 2015–2019

Between 2015 and 2019, 303 hospitalized patients with scrub typhus were admitted. The heat map statistics showed that there were two incidence peaks of scrub typhus in the northern region of Fujian. The prominent epidemic peak was from June to July each year, and the minor peak was from October to November each year. There were almost no cases from January to April each year, which belonged to this region's static phase of the scrub typhus epidemic, indicating that the disease had typical seasonality (Fig. 4).

### Typical eschar distribution

**Fig. 5:**
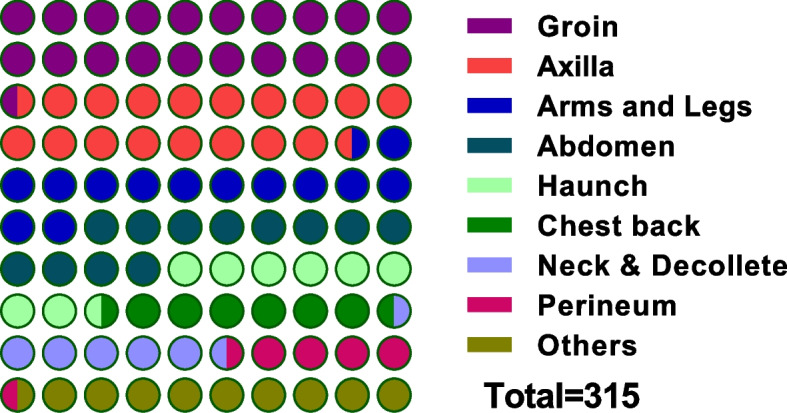
The distribution of eschar in 303 patients with scrub typhus. The circle diagram shows the proportion of eschar distribution in different parts. One hundred circles represent the percentage; two-color circles will appear if the proportion is not an integer. The specific proportion of each part is as follows: Groin (20.64%), Axilla (18.10%), Arms and legs (13.33%), Abdomen (12.06%), Hanch (8.57%), Chest back (6.67%), Neck and Decollete (6.35%), Perineum (4.76%), and Others (9.52%)

The characteristic eschar was found in all 303 patients with scrub typhus included in this study, with a total of 315 eschar sites (a few patients had multiple eschar lesions), including 65 (20.64%) in the groin, 57 (18.10%) in the axilla, 42 (13.33%) in the limbs, 38 (12.06%) in the abdomen, and others including the breast, penis, scalp, perianal, and other sites. It can be seen that the proportion of eschar appearing in the groin and axilla is the most common, and the incidence is significantly higher than in other parts (Fig. 5).

### Clinical manifestations and complications

The main clinical manifestations of 303 scrub typhus were fever (97.69%), fatigue (76.24%), muscle soreness (66.34%), and headache (76.24%). The main signs were eschar (100%), lymphadenopathy (70.63%), and splenomegaly (31.68%) (Table 2). There were 209 patients (69.97%) with various complications, and the main complications included acute liver injury (57.75%), electrolyte metabolism disorder (33.66%), multiple organ function injury (32.34%), thrombocytopenia (29.37%), and pulmonary infection (23.43%) (Table 3). The sample size of pediatric patients was small (*n* = 12); therefore, they were not compared with adult patients. The main clinical manifestations in these patients were fever (91.67%), eschar (100%), fatigue (75%), headache (58.33%), cough (50%), lymphadenopathy (66.67%), splenomegaly (33.33%) and pulmonary rales (25%). There were no severe cases in any of the children, mainly related to parents' attention to children's health. All children were treated early, and the prognosis were good.

**Table 2:** The clinical symptoms and signs of 303 cases of scrub typhus

Symptoms	n	%	Signs	n	%
Fever	296	97.69	Eschar	303	100.00
Fatigue	231	76.24	Lymphadenectasis	214	70.63
Muscle soreness	201	66.34	pulmonary rales	133	43.89
Headache	196	64.69	Splenomegaly	96	31.68
Cough & expectoration	141	46.53	Hepatomegaly	92	30.36
Chills	125	41.25	Rash	81	26.73
Nausea & vomiting	97	32.01	Jaundice	78	25.74
Chest pain	32	10.56	conjunctival congestion	52	17.16
Dyspnea	22	7.26	Bleeding spots	41	13.53
Hemoptysis	20	4.62	Heart murmurs	6	2.31
Shock	12	3.96	Meningeal irritation sign	5	1.65
Hematochezia	6	1.98	Peritoneal irritation sign	3	0.99

**Table 3:** Complications in hospitalized patients

Complications	n	Constituent ratio (%)
Acute liver injury	175	57.75
Electrolyte metabolism disorders	102	33.66
Mutiple Organ Disfunction	98	32.34
Thrombocytopenia	89	29.37
Acute kidney injury	85	28.05
pulmonary infections	71	23.43
Dyslipidemia	68	22.44
Anemia	56	18.48
Hypoproteinemia	46	15.18
Acute gastritis	18	5.94
Hyperfibrinogen	17	5.61
Septic shock	15	4.95
Myocarditis	11	3.63
Respiratory failure	10	3.30
Acute cholecystitis	7	2.31
Gastrointestinal hemorrhage	6	1.98
Meningoencephalitis	5	1.65
DIC	2	0.66

### Laboratory test results

Routine blood, biochemical test, coagulation function, inflammation index, and urine routine were analyzed in 303 patients, and the results are shown in Table 4. Before diagnosis, all patients routinely underwent the Weil-Felix test, and the submission rate was 100%. No repeated submission was made, and the specimens were sent within 1–20 days of the course of the disease, among which 17 cases were OXK-positive (The OXK titer ≥ 1:160), with a positive rate of 5.61% (Table 4).

**Table 4:** Laboratory test results

Test	Number	Constituent ratio (%)
**Blood RT**		
Leucopenia ( WBC < 4 × 10^9^/L)	164	54.13
Leukocytosis ( WBC > 10 × 10^9^/L)	91	30.03
Eosinophil count decreased(E < 0.5 × 109/L)	156	51.48
Neutrophils increased(N > 75%)	84	27.72
Thrombocytopenia ( PLT < 100 × 109/L)	89	29.37
Hemoglobin dropped(Hb < 120 g/L)	56	18.48
**Chem-Bio Detection**		
Serum creatinine increased(Cr > 90 μmol/L)	65	21.45
Alanine aminotransferase increased(ALT > 40 U/L)	175	57.75
Total bilirubin increased(TB > 20 μmol/L)	79	26.07
Creatine kinase increased(CK > 167 U/L)	107	35.31
**Weil-Felix test**		
OXK (≥ 1:160)	17	5.61
**Coagulation function**		
Prothrombin time prolonged (> 14.5 s)	49	16.17
D-dimer (> 200 μg/L)	46	15.18
**Urinalysis**		
U-WBC (male > 3/hp, female > 5/hp)	21	6.93
Urine occult blood (> + ~ ~ + + +)	52	17.16
Urine protein (> + ~ ~ + + +)	134	44.22
**Inflammation index**		
C-reactive protein increased(CRP > 10 mg/L)	296	97.69
Procalcitonin (> 0.5ug/L)	37	12.21
ESR ( male > 15 mm/h, female > 20 mm/h)	102	33.66

(1) *Blood RT* Blood routine test, *WBC* White blood cell, *E* Eosinophil, *N* Neutrophils, *PLT* Thrombocytopenia, *Hb* Hemoglobin, *Cr*,Serum creatinine, *ALT* Alanine aminotransferase, *TB* Total bilirubin, *CK* Creatine kinase, *U-WBC* Urine white blood cell, *CRP* C-reactive protein, *ESR* Erythrocyte sedimentation rate

### Prognosis and outcome

**Fig. 6:**
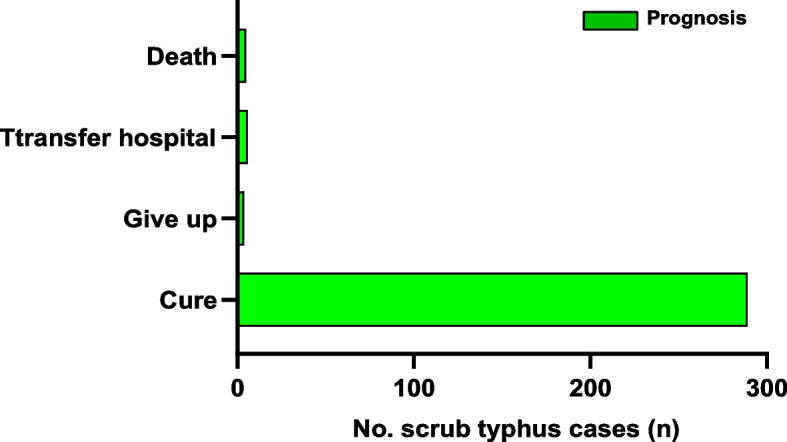
The Prognosis of 303 scrub typhus patients

Scrub typhus is easily missed or misdiagnosed, and the misdiagnosis rate is high when non-specialist doctors visit. It is easily misdiagnosed as pulmonary infection, sepsis, or fever of unknown origin. The First Hospital of Nanping is the largest tertiary hospital in Northern Fujian. Patients with poor treatment effects in other counties and cities were referred to the hospital, and 145 had used antibiotics in other hospitals. Before being diagnosed, such transfer patients had been treated with second- and third-generation cephalosporins, quinolones, carbapenems, and other antibiotics, but the efficacy was not apparent. After systematic physical examination, medical history collection, and laboratory testing by infectious physicians, anti-rickettsial treatment with chloramphenicol or doxycycline was administered. The treatment course lasted 1–2 weeks. All 289 patients were cured after anti-rickettsial and symptomatic treatment. The main reasons for poor prognosis include misdiagnosis, delayed application of effective anti-rickettsial therapy, multiple organ failure, underlying diseases, and other factors (Fig. 6). This study also compared the gender, age, occupation, residence, and diagnosis time between the cured and uncured groups, and found that the prognosis was related to non-farmer occupation and diagnosis time ≥ 8 d (Table 5).

**Table 5:** Correlative factor associated with prognosis from scrub typhus, Nanping, 2015–2019

Factor	Cured cases	Uncured cases	Chi-square	95%CI	P
**Sex**					
Male	143	8	0.314	0.452–4.042	0.5755
Female	146	6			
**Residence**					
Rural	198	11	0.631	0.503–5.755	0.427
Urban	91	3			
**Occupation**					
Farmer	225	7	5.775	0.106–0.7702	0.016*
Nonfarmer	64	7			
**Age groups (y)**					
0–9	11	0	NA	NA	NA
10–19	4	0	NA	NA	NA
20–29	14	0	NA	NA	NA
30–39	22	0	NA	NA	NA
40–49	51	1	1.036	0.033–2.344	0.309
50–59	69	4	0.161	0.429–3.923	0.688
60–69	75	6	1.948	0.709–6.521	0.163
70–79	40	2	0.002	0.224–4.403	0.963
˃80	3	1	3.803	0.525–51.15	0.051
**Diagnosis time**					
< 3	202	0	NA	NA	NA
3–7	59	3	0.008	0.276–3.237	0.927
≥8	28	11	56.50	9.334–117.1	< 0.0001*

^*^ indicates significance (*p* < 0.05). NA is not available

## Discussion

Scrub typhus is a natural epidemic caused by Orientia tsutsugamushi. It is thought to affect one million people annually, and it is believed that approximately one billion individuals are at risk of contracting it annually. The spread of scrub typhus closely follows the distribution pattern of the rodent species Rattus rattus [12]. Scrub typhus, also known as tsutsugamushi disease, is brought on by Orientia tsutsugamushi, one of the oldest known vector-borne pathogens worldwide. This strictly intracellular gram-negative bacterium requires host cells and infects people through the bites of chigger mite larvae. Mite species that are crucial as O. tsutsugamushi potential vectors are generally found throughout the world. Scrub typhus currently does not have a vaccine, so general preventative measures include avoiding exposure, dressing appropriately, and using repellents to avoid chigger mite bites [1, 13]. Due to the lack of specificity of clinical symptoms, scrub typhus is easily misdiagnosed. Currently, no vaccine is available to prevent the incidence of scrub typhus. This study describes the epidemiological distribution of scrub typhus in northern Fujian to understand the incidence characteristics and influencing factors in this area. To provide a scientific basis for preventing and controlling scrub typhus and developing measures to reduce the public health burden and promote public health.

By 2021, the area had a permanent population of 2.67 million, making it the principal endemic location for scrub typhus in Fujian Province. In northern Fujian, Yanping District, Shunchang County, Zhenghe County, and Songxi County had the highest number of scrub typhus cases, all at a high level according to the local population. Clinical data of 303 patients with scrub typhus were retrospectively analyzed. It was found that 151 (49.83%) were males and 152 (50.17%) were females. There was no significant difference in the number of hospitalized patients between males and females. The patients mainly concentrated between 40–49 years old (17.16%), 50–59 years old (24.09%), and 60–69 years old (26.73%). Age clustering was related to occupation, and the high-risk population had more frequent field activities, especially farmers who had been engaged in agriculture for a long time (76.57%). Due to the relatively backward economy in northern Fujian, the primary economic source is agriculture, and most farmers are over 40 years old. The population in this area is aging significantly, and the young and middle-aged population flows out all year round, so the proportion of young and middle-aged patients is low. Our results were consistent with previous studies, indicating no significant change in scrub typhus's high-risk population, age, occupation, and population characteristics [14]. In terms of occupation, consistent with the survey in neighboring provinces, farmers were also a high-risk group in northern Fujian because of increased exposure to pathogen-carrying chigger mites and insufficient protection during planting, harvesting, or other field agricultural activities [15, 16]. In addition, although it is considered a rural disease, this study found that the proportion of patients in urban areas is not low, and 94 (31.03%) cases lived in urban areas for a long time. This may be related to their greater leisure time devoted to outdoor recreational activities, such as walking in the park. The literature has reported that the eco-friendly trend of having more natural parkland and gardens within the city may create more suitable habitats for vectors and rodent hosts [17].

The southeast coastal areas of our country are characterized by high temperatures and high humidity, which are suitable for the breeding and reproduction of microorganisms, animals, and insects. They were also specific foci of scrub typhus, and the coastal island foci were mainly distributed in Fujian Province. Nanping is located in the north of Fujian province; mountainous areas and hills are widely distributed in the subtropical humid monsoon climate. It has jurisdiction over two municipal districts, five counties, and three county-level cities, covering an area of 26,300 square kilometers, ranging from 117°00 'E to 119°25' E and from 26°30 'N to 28°20' N [14, 18, 19]. In recent years, the People's Government of Nanping City has actively responded to national policies and vigorously carried out ecological civilization construction, such as restoring forests and wetlands, banning the traditional burning of straw in rural areas, and reducing industrial land use. This also increases the suitability of the environment for rodents and chigger mites while increasing the possibility of human exposure [20]. Nanping City is located in the north of Fujian Province. The main terrain is hilly and mountainous, with dense forests, shrubs, weeds, mountain streams, and rivers all over the upper reaches of the Minjiang River. The external environment is suitable for the reproduction of chigger larval mites. Among the ten counties and cities in this area, the terrain of Jianyang and Jian 'ou is dominated by plains and hills, and hills and mountains dominate those of other counties and cities. The results of the SDE map showed that the distribution of scrub typhus cases in northern Fujian was in a specific direction, mainly from Yanping, Shunchang, Zhenghe, and Songxi counties. In conclusion, habitat complexity and diversity of land cover are essential drivers of disease emergence. Therefore, we conclude that it is necessary to focus on prevention and control in these four areas and strengthen health education.

Seasonal decomposition analysis indicated that scrub typhus cases were highest in summer, followed by autumn [14]. From 2015 to 2019, there were two incidence peaks of scrub typhus in northern Fujian; the prominent epidemic peak was from June to July each year, and the secondary epidemic peak was from October to November. There were almost no cases from January to April each year, which belonged to the quiescent period of the scrub typhus epidemic in this area, indicating that the outbreak of scrub typhus had a typical seasonal pattern in this area [21]. Critical factors for scrub typhus transmission are ambient temperature, humidity, and rainfall, with climatic conditions from June to November, and abundant food during the harvest season is best suited for rodent host survival. Moreover, summer and autumn are the seasons in which humans live outdoors for the longest time, so the occurrence of the disease has a seasonal appearance [22]. Therefore, it is best to wear long pants and long-sleeved clothes in summer and autumn to avoid exposing the grass for a long time and to reduce the chance of chigger bites.

Diagnosing and treating this disease is not timely; severe complications may occur, and even life-threatening, so primary doctors should improve their understanding of this disease [23]. The primary pathological changes in scrub typhus are systemic minor vessel inflammation and perivascular inflammation, which cause congestion, edema, cell degeneration, and necrosis of solid organs [24]. It can involve multiple systems and organs, and its diverse clinical manifestations make it easy to misdiagnose [25]. This study included 303 patients with scrub typhus, and 97.69% had fever as the first diagnosis. The most common clinical symptom of scrub typhus is fever, which is characterized by acute and high fever. Therefore, fever as a disease has little specificity and is most easily misdiagnosed as a respiratory tract infection [19]. Eschar or ulcer is a specific sign of scrub typhus. However, its location is hidden and difficult to detect, and it is easy to miss a diagnosis without careful physical examination [26]. In this study, eschar ulcers were mainly distributed in the groin and axilla, with two eschar sites observed in some cases. The results also suggest that the incidence of lymphadenopathy is lower than fever and eschar, which is a common sign. In this study, characteristic eschar was found in all 303 cases of scrub typhus, and 315 eschar sites were found.

Among them, the groin and axilla accounted for the highest proportion, mainly due to the warm and humid environment of chigger mites. Lymphadenopathy can be used as a diagnostic clue for scrub typhus. The incidence of rash is lower than that in the literature, a characteristic of scrub typhus in Fujian province, and may also be related to the short duration of treatment and rash [27]. This study found many complications of scrub typhus involving multiple organs, among which liver function damage was the most common (57.75%), and most were mild to moderate. Biochemical examination showed that ALT levels exceeded the expected value in 175 cases. Renal function impairment (28.05%) is common in patients with scrub typhus, and it has been reported that renal function impairment in Fujian is higher than in other areas. Whether this is related to the epidemic serotype requires further investigation. It is worth noting that patients with scrub typhus in this region often have positive urinary protein combined with abnormal renal function and thrombocytopenia, which is often misdiagnosed as hemorrhagic fever with nephrotic syndrome [28]. Therefore, it is essential to conduct a systematic physical examination of patients with fever, especially to identify the characteristic eschar, which is helpful for the diagnosis of the disease.

Laboratory results suggested that scrub typhus could affect multiple system functions, including the blood system, liver function, kidney function, coagulation function, and various inflammatory indicators [29]. In this study, 54.13% of the patients with scrub typhus had reduced white blood cell count, and 51.48% had reduced eosinophil counts, suggesting that white blood cell and eosinophil levels can be used as an auxiliary examination method. This and other studies have reported a high incidence of thrombocytopenia, which is easily misdiagnosed as a blood system disease, and thrombocytopenia can also be used as an additional examination feature of scrub typhus. The positive rate of the Wei-Fei test is low, and it is easy to miss and delay the diagnosis. The only specific laboratory diagnostic test for scrub typhus in northern Fujian Province is the Weil-Felix test. In this study, 303 patients were tested; only 17 cases were positive, indicating a higher positive rate. The reasons for the low positive rate of this test may be as follows: (1) The Weil-Felix test is a Proteus agglutination reaction established by using the cross immunogenicity of *Orientia tsutsugamushi* and *Bacillus proteus*, so its sensitivity and specificity are not high [30]; (2) because of the complex composition of scrub typhus serum, the antibody appeared late, and it was not easy to detect in the early stage; (3) there is a particular relationship with the selected experimental reagent. If scrub typhus is suspected, a scrub typhus-specific antibody test or next-generation sequencing detection technology (NGS) should be performed as soon as possible [31]. Therefore, clinicians should not rely on laboratory tests to diagnose tsutsugamushi disease but should combine patients' epidemiology and clinical manifestations to avoid the delay of the disease.

The prognosis of scrub typhus is closely related to the timely diagnosis. Inexperienced doctors are often prone to missed diagnoses or misdiagnoses, so it is necessary to strengthen their training in relevant knowledge. All 289 cases were cured after anti-rickettsial and symptomatic treatment. The main reasons for poor prognosis include misdiagnosis, delayed application of effective anti-rickettsial therapy, multiple organ failure, underlying diseases, and other factors. This study also compared the gender, age, occupation, residence, and diagnosis time between the cured and non-cured groups and found that the prognosis was related to non-farmer occupation and diagnosis time ≥ 8 d. Farmers may have higher immunity from long-term physical work, while non-farmers have lower immunity due to lack of physical exercise, so the prognosis is poor. Most of the cases in this project visited the Department of Infection of the First Hospital of Nanping City due to unexplained fever or abnormal liver function. Most patients had a history of treatment in primary health centers and were referred to our hospital due to unknown etiology and the long course of the disease. Therefore, patients with unexplained fever must carefully do a physical examination and other relevant clinical symptoms to investigate whether they have scrub typhus. The etiological treatment of scrub typhus is relatively simple, and doxycycline and chloramphenicol are effective [32]. The death cases in this study were mainly due to early misdiagnosis and delayed etiological treatment, complications, and multiple organ failure, leading to death [33]. Therefore, improving the understanding of scrub typhus, reducing misdiagnosis, and improving early diagnosis is the key to treatment.

## Conclusions

Knowledge of the geographical distribution and burden of scrub typhus is critical to determine the best allocation of limited resources for disease prevention and control. Simultaneously, clinicians should enhance awareness of nonspecific fever symptoms among residents in high-risk locations to diagnose scrub typhus early and more precisely. In addition to contributing to the scientific advancement of scrub typhus epidemiology in Fujian Province, the findings of our study provide critical evidence to health authorities, policymakers, public health practitioners, and other service providers in developing feasible strategies and issuing targeted guidelines.

This study has limitations and lacks multi-center and large sample size clinical data studies. Accurately estimating the prevalence of scrub typhus in this region because of the lack of data on all patients in lower medical institutions. We will further contact subordinate medical institutions to comprehensively analyze the epidemiological characteristics of scrub typhus in this region. The relationships between potentially influential factors in climatic, geographical, and environmental aspects and scrub typhus incidence in Nanping City will be further explored.

## Data Availability

The datasets used and analyzed in this study belong to our research team and do not include personal privacy information. The datasets are available from the corresponding author upon request.

## Abbreviations

DIC: Disseminated intravascular coagulation
BRT: Blood routine test
WBC: White blood cell
CRP: C-reactive protein
PCT: Procalcitonin
E: Eosinophil
N: Neutrophils
PLT: Thrombocytopenia
Hb: Hemoglobin
Cr: Serum creatinine
TB: Total bilirubin
ALT: Alanine aminotransferase
CK: Creatine kinase
U-WBC: Urine white blood cell
ESR: Erythrocyte sedimentation rate
ST: Scrub typhus
SDE: Standard deviation ellipse
NGS: Next-generation sequencing detection technology
